# The potential role of Saiko‐ka‐ryukotsu‐borei‐to in mitigating the severity of anxiety induced by social defeat stress in mice

**DOI:** 10.1002/pcn5.70191

**Published:** 2025-08-19

**Authors:** Yoshikazu Kitai, Leo Gotoh, Hikaru Hori

**Affiliations:** ^1^ Department of Psychiatry, Faculty of Medicine Fukuoka University Fukuoka City Japan; ^2^ Laboratory of Neuroscience, Department of Psychiatry, Faculty of Medicine Fukuoka University Fukuoka City Japan

**Keywords:** anxiety, fluoxetine, mitigation, Saiko‐ka‐ryukotsu‐borei‐to, social defeat stress

## Abstract

**Aim:**

Major depressive disorder is a growing global concern with limited treatment options. Social stress contributes to its development, yet pharmacological prevention and mitigation remain underexplored. This study examined the effects of Saiko‐ka‐ryukotsu‐borei‐to (SRBT) on depressive and anxiety‐like behaviors in mice exposed to social defeat stress (SDS).

**Methods:**

C57BL/6J mice were subjected to daily 10‐min interactions with larger, more aggressive ICR mice for 10 consecutive days to induce SDS. Immediately following each session, mice were orally administered SRBT, fluoxetine (Flu), or saline (Sal), and were assigned to the SD‐SRBT, SD‐Flu, and SD‐Sal groups, respectively. Mice that received Sal without SDS exposure served as the normal control (NC) group. On the 11th day, behavioral assessments, including the elevated plus maze (EPM) test, tail suspension test (TST), and social interaction test (SIT), were conducted across the four groups. Plasma corticosterone levels were also measured.

**Results:**

SDS significantly increased anxiety‐like behavior in the EPM, as shown by reduced open arm time in the SD‐Sal and SD‐Flu groups. This effect was less evident in the SD‐SRBT group. Although the SD‐Flu group showed similar anxiety‐like behavior to the SD‐Sal group, no significant difference in open arm time was observed. SDS did not induce social avoidance or depressive‐like behavior in the SIT or TST, nor did it alter plasma corticosterone levels.

**Conclusion:**

This study suggested that SRBT has the potential to mitigate anxiety caused by social stress. However, further ongoing evaluation and investigation are required to assess the effectiveness of SRBT.

## INTRODUCTION

Major depressive disorder (MDD) is a prevalent psychiatric disorder globally,^1^, ^2^ and it imposes a significant economic burden.^3^, ^4^ In addition, MDD leads to significant impairments of quality of life,^5^ and thus, appropriate treatment and prevention options are desired, along with interventions for the mitigation of progression to more severe forms. Antidepressants and psychotherapy, as the usual treatments for MDD, have limited efficacy.^6^, ^7^ Although many MDD treatment guidelines have been reported,^8^, ^9^, ^10^, ^11^ current treatments reduce the overall disease burden by only one‐third.^12^ In addition, only a small fraction of patients with MDD receive adequate treatment.^13^

Several factors contribute to the development of MDD, including the hypothalamus–pituitary–adrenal medulla (HPA) system, monoamines, inflammation, psychosocial functioning, brain dysfunction, and genetic–epigenetic factors.^14^ Of these, stress is the most well‐known factor in the pathogenesis of MDD. Social stressors are known to be particularly important.

Several reports have described the prevention of MDD through psychotherapy and lifestyle. For example, improved sleep, diet,^15^ exercise,^16^, ^17^, ^18^ social interventions, and psychotherapy have been reported to be effective.^19^ Reports have also detailed the effectiveness of cognitive behavioral therapy^20^ and educational interventions. Meanwhile, few studies have examined the efficacy of pharmacotherapy in preventing MDD, including the mitigation of its progression to more severe stages.

Saiko‐ka‐ryukotsu‐borei‐to (SRBT) is a medicine comprising 11 herbs. This prescription has been recommended for several neuropsychiatric conditions, including anxiety, neurosis, and irritability. A meta‐analysis revealed the effectiveness of SRBT against poststroke depression.^21^ In our previous study, SRBT also improved depression‐like behavior in an olfactory bulbectomized model of depression in rats.^22^ However, the efficacy of SRBT in preventing MDD, including the mitigation of its progression to more severe forms, remains unclear.

Therefore, this study evaluated the mitigation effects of SRBT on depressive and anxious behaviors induced by social defeat stress (SDS) in C57BL/6J mice, using behavioral analyses—including the social interaction test (SIT), tail suspension test (TST), and elevated plus maze (EPM) test—as well as assessments of plasma corticosterone levels.

## MATERIALS AND METHODS

### Animals

Male C57BL/6J and ICR mice were purchased from Japan SLC, Inc. (Shizuoka, Japan) and used at 6 and 10–12 weeks old, respectively. Food and water were provided ad libitum. The vivarium was maintained at 23°C ± 1°C under a 12‐h/12‐h light/dark cycle (lights on at 8:00 a.m.). The research protocol was approved by the Fukuoka University Ethical Review Board on Animal Testing (approval number: 2409042).

### Drugs

SRBT (Tsumura Co., Ltd., Tokyo, Japan) was dissolved in saline and administered orally to C57BL/6J mice at a dose of 300 mg/kg, in line with a previous experiment.^23^ Flu (Sigma‐Aldrich, St. Louis, MO, USA) was dissolved in Sal and administered orally to C57BL/6J mice at a dose of 10 mg/kg, as previously reported.^24^

### SDS

Mice were exposed to SDS using previously described methods.^25^ Specifically, C57BL/6J mice were stressed by being placed in the home cage of an aggressive ICR mouse daily 10 min for 10 consecutive days. To minimize the impact of variability in the aggressiveness of the ICR mice, the pairs of C57BL/6J and ICR mice were randomized daily. After stress exposure, C57BL/6J mice were returned to their original home cages. C57BL/6J mice were visually observed to identify defensive behaviors, such as escape or submissive postures, in response to physical attacks by ICR mice, which served as an indicator that SDS had been successfully established. It was visually confirmed that C57BL/6J mice exhibited defensive behaviors such as avoidance and submissive postures in response to physical attacks by ICR mice, indicating that the C57BL/6J mice were defeated by the ICR mice.

### Experimental design

**Figure 1:**
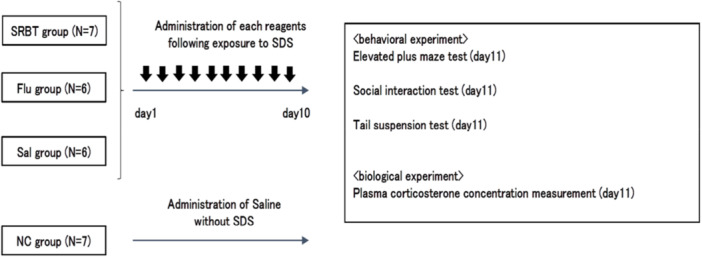
Schematic representation of the experiment design. Flu, fluoxetine; NC, normal control; Sal, saline; SDS, social defeat stress; SRBT, Saiko‐ka‐ryukotsu‐borei‐to.

C57BL/6J mice exposed to SDS were divided into three groups based on treatment with SRBT (*N* = 7), Flu (*N* = 6), or Sal (*N* = 6). Additionally, C57BL/6J mice that were administered Sal without being exposed to SDS comprised the normal control (NC) group (*N* = 7). The treatments were administered immediately after stress exposure to evaluate the stress‐mitigating effects of each intervention by targeting the early phase of the stress response process. This experimental procedure was repeated for 10 consecutive days. On Day 11, behavioral tests were conducted, followed by euthanasia under inhalation anesthesia with isoflurane (FUJIFILM Wako Pure Chemical, Osaka, Japan) for biological experiments (Figure 1).

C57BL/6J mice exposed to SDS were divided into three groups based on treatment with SRBT (*N* = 7), Flu (*N* = 6), or Sal (*N* = 6). Additionally, C57BL/6J mice that were administered Sal without being exposed to SDS comprised the NC group (*N* = 7). The treatments were administered immediately after stress exposure to evaluate the stress‐mitigating effects of each intervention by targeting the early phase of the stress response process. This experimental procedure was repeated for 10 consecutive days. On Day 11, 24 h after the final drug administration on Day 10, behavioral tests were conducted in the order of SIT, EPM, and TST, with approximately 30‐min intervals between each test. Following behavioral assessment, mice were euthanized under inhalation anesthesia with isoflurane (FUJIFILM Wako Pure Chemical, Osaka, Japan) for the collection of blood samples intended for subsequent biological experiments. Blood sample collection was scheduled on Day 11 to avoid acute stress responses that may confound the evaluation of sustained effects induced by SDS exposure and pharmacological interventions.

### Behavioral assays

#### TST

The TST was used to measure depression‐associated behavior. This procedure was based on a previous experiment.^26^ The tail of the mouse was fixed to a rod installed in the test device, which allowed the mouse to be hung upside‐down. The immobility time during the 5‐min test session was measured and analyzed using Panlab SMART ver. 3.0.06, and the proportion of immobility was assessed.

#### EPM test

The EPM test was used to measure anxiety‐associated behaviors. The procedure was based on a previous experiment.^27^ The device consisted of an open arm (50 × 10 cm), a closed arm with a wall (50 × 10 × 40 cm), and a central square (10 × 10 cm) at a height of 60 cm. The time spent in the open arm was analyzed using Panlab SMART ver. 3.0.06.

#### SIT

The SIT was used to measure social behavior in terms of social proximity in mice. This procedure was based on a previous experiment.^28^ First, the mice were placed in an open field (30 × 30 × 40 cm) with empty SIT cages and allowed to explore freely for 5 min. For the next 5 min, the mice were allowed to explore freely in an open field (30 × 30 × 40 cm) containing SIT cages together with an unfamiliar mouse. The area close to the SIT cages was designated as the high interaction zone. The movements of the mice during the first 5 min and the next 5 min were compared and analyzed using Panlab SMART ver. 3.0.06.

### Biochemical analysis of plasma corticosterone levels

The mice were sacrificed by decapitation, and their trunk blood was collected in a hematocrit capillary with heparin tubes. Blood samples were centrifuged at 3000 rpm for 15 min and stored at −80°C. Plasma corticosterone levels were determined using an enzyme immunoassay kit (Cayman Chemical Company, Ann Arbor, MI, USA) according to the manufacturer's protocol.

### Statistical analysis

Analysis of variance (anova) and Turkey–Kramer's honest significant difference test were used to compare the groups. Statistical significance was set at *p* < 0.05. All statistical analyses were performed using JMP12.2.0 (SAS Institute Inc., Cary, NC, USA).

## RESULTS

### TST

**Figure 2:**
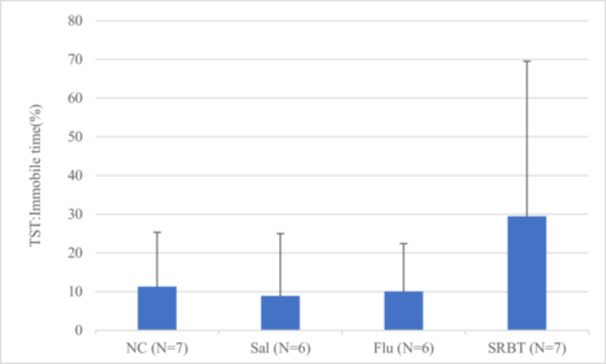
Ratio of immobile time in the tail suspension test (TST). The results are presented as the percentage of time during which the mice were immobile during the 5‐min TST. Data are presented as the mean ± SEM. *p < 0.05. Flu, fluoxetine administration in mice with social defeat stress; NC, normal control; Sal, saline administration in mice with social defeat stress; SRBT, Saiko‐ka‐ryukotsu‐borei‐to administration in mice with social defeat stress.

anova revealed no significant difference among the groups regarding the percentage of time during which mice were immobile in the TST (*p* = 0.5755, Figure 2).

### EPM test

**Figure 3:**
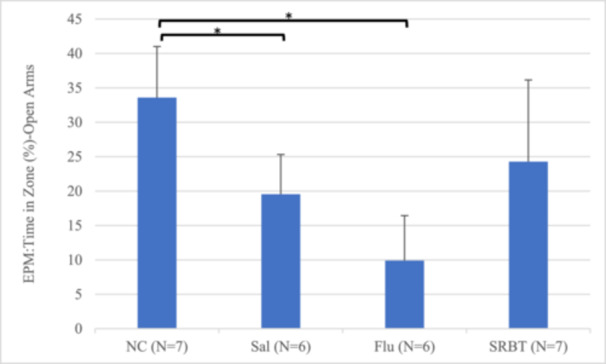
Ratio of time spent in the open arms of elevated plus maze (EPM) test. The results are presented as the percentage of time spent in the open arms over a 5‐min period. Data are presented as the mean ± SEM. *p < 0.05. Flu, fluoxetine administration in mice with social defeat stress; NC, normal control; Sal, saline administration in mice with social defeat stress; SRBT, Saiko‐ka‐ryukotsu‐borei‐to administration in mice with social defeat stress.

anova revealed significant differences in the percentage of time spent in the open arms among the groups (*p* = 0.0028). The percentage of time spent in the open arms was significantly lower in mice exposed to SDS and then treated with saline (Sal) or fluoxetine (Flu) than in NC mice (*p* = 0.0339 and *p* = 0.0097, respectively, Figure 3).

### SIT

**Figure 4:**
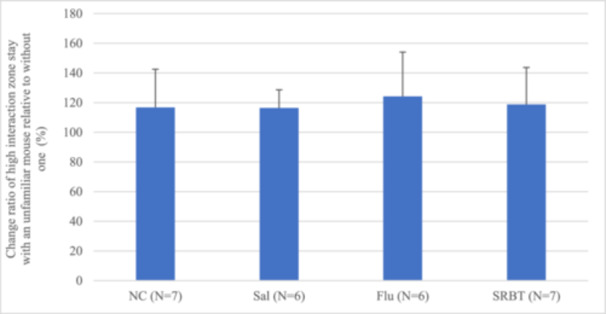
Social interaction test (SIT) rate of change in the high interaction zone stay ratio. Results from SIT. Ratio of change in the high interaction zone stay ratio with an unfamiliar mouse in the social interaction cage relative to that without mouse. Data are presented as the mean ± SEM. *p < 0.05. Flu, fluoxetine administration in mice with social defeat stress; NC, normal control; Sal, saline administration in mice with social defeat stress; SRBT, Saiko‐ka‐ryukotsu‐borei‐to administration in mice with social defeat stress.

anova revealed no significant difference between groups regarding the percentage of time spent in the center area (*p* = 0.9945, Figure 4).

### Plasma corticosterone levels

**Figure 5:**
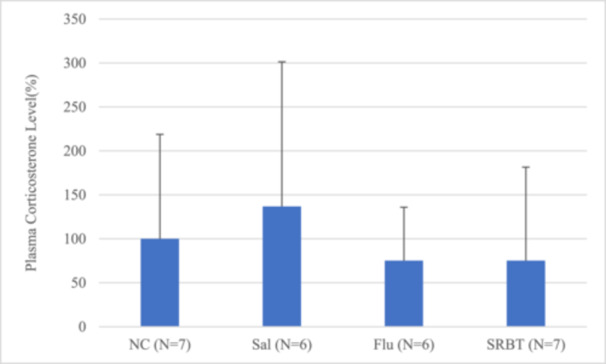
Plasma corticosterone levels. Plasma corticosterone levels, as measured using an enzyme immunoassay kit, are presented relative to those in the normal control (NC) group (set at 100%). Data are presented as the mean ± SEM. *p < 0.05. Flu, fluoxetine administration in mice with social defeat stress; Sal, saline administration in mice with social defeat stress; SRBT, Saiko‐ka‐ryukotsu‐borei‐to administration in mice with social defeat stress.

anova revealed no difference in plasma corticosterone levels among the groups (*p* = 0.7871, Figure 5).

## DISCUSSION

In the present study, we significantly increased anxiety in C57BL/6J mice by exposing these mice to ICR mice for 10 min/day for 10 consecutive days, thereby inducing SDS. However, C57BL/6J mice orally administered 300 mg/kg SRBT immediately after SDS did not exhibit a significant increase in anxiety, as observed in the SD‐Sal group. This study suggests that administering SRBT immediately after inducing SDS may alleviate the anxiety enhancement caused by SDS. Meanwhile, the results of the EPM test indicated that in the SD‐Flu group, an increase in anxiety‐like behavior, similar to that observed in the SD‐Sal group, was noted; the average time spent in the open arms of the EPM test by the SD‐Flu group tended to be lower compared to the SD‐Sal group, but no significant difference was observed. Therefore, it could not be concluded that the administration of Flu alleviated the anxiety enhancement caused by SDS.

In a previous study,^29^ rats were exposed to “nonsocial” stress for 2 h daily through transfer to a wire‐net stress cage with immersion in a water bath maintained at 21°C to the level of the xiphoid process for 28 days. This was followed by a 10‐day recovery period, during which 1000 mg/kg SRBT, 10 mg/kg diazepam, or 10 mL/kg distilled water was administered daily. Then, 24 h after the last dose (Day 39), the time spent in the open arm of the EPM test was measured. The results illustrated that chronically stressed rats spent significantly less time in the open arm than unstressed rats. In addition, treatment with 1000 mg/kg SRBT after chronic stress exposure significantly reverses the shortening of the open arm residence time in the EPM test. Differing from this prior study, our study utilized mice instead of rats, the dose of SRBT was lower, and mice were exposed to social stress opposed to chronic stress. Our study suggests that administering SRBT immediately after exposure to SDS may help mitigate related anxiety symptoms.

In a study investigating the effects of Flu on anxiety‐ and depression‐like behavior,^30^ C57BL/6J mice (male, 5 weeks old) were exposed to 6 h/day restraint stress for 5 consecutive days in a transparent cylinder composed of plexiglass with small holes (with a flat bottom and a stopper supporting the rear of the animal). After stress exposure, mice were administered 20 mg/kg Flu or vehicle for 5 consecutive days. Flu significantly increased the time spent in the open arm in the EPM test versus vehicle. In a prior study, 5‐week‐old C57BL/6J mice were utilized, exposed to a nonsocial stress paradigm, and administered a high dose of fluoxetine. In contrast, our study utilized 8‐week‐old mice, implemented a social stress model, and administered a lower dose of fluoxetine. Another study systematically examined the effects of selective serotonin reuptake inhibitors (SSRIs) on various naturally occurring anxieties in animal models,^31^ finding that SSRIs significantly reduced anxiety‐like behavior in the EPM test. However, the effects of SSRIs on anxiety‐like behavior were dependent on the disease state of the animals. In our study, fluoxetine was administered to stressed mice; however, no anxiolytic effects were observed.　Specifically, the anxiolytic effect of SSRIs was significantly greater in stressed subjects than in nonstressed animals. Conversely, another study reported that Flu destabilizes emotions in mice.^32^ In this study, C57BL/6J mice treated with 14 mg/kg Flu for 4 weeks displayed no significant changes in the EPM test compared with the findings in the control group, whereas mice treated with 22 mg/kg Flu for 4 weeks displayed a significantly reduced number of entries into the open arm and a numerical reduction in the time spent in the open arm. In other words, anxiety was enhanced in C57BL/6J mice by the higher dose of Flu. In our study, stress was applied to mice, a lower dose of fluoxetine was administered, and anxiety‐like behavior was similarly enhanced. Based on these findings, the anxiolytic effects of fluoxetine depend on multiple factors, including the presence or absence of stress exposure, the type of stress, the dose of fluoxetine, and the duration of its administration. Under certain conditions, fluoxetine may fail to exhibit its effects or even enhance anxiety‐like behavior. In humans, symptoms such as hyperactivity, irritability, insomnia, disinhibition, anxiety, and increased excitability (activation syndrome) rarely occur in humans treated with SSRIs.^33^ Although many studies have found that fluoxetine alleviates anxiety, a small number have reported that the drug may instead enhance anxiety. In our study, while a significant difference was observed between the NC and SDS‐Flu groups, no significant difference was found between the SDS‐Sal and SDS‐Flu groups. Therefore, our findings do not provide clear support for either interpretation.

In our study, we observed no significant differences in the SIT, TST, or plasma corticosterone levels—partially diverging from findings reported in a previous study.^25^ Both investigations utilized the C57BL/6 mouse strain and adopted a 10‐day SDS protocol involving daily 10‐min physical interactions. Additionally, similar schedules for behavioral assessment were employed, with neither study detecting depression‐like phenotypes. Notable methodological differences, however, may account for the observed discrepancies. The prior study used 24‐day‐old male C57BL/6L mice and ICR aggressors aged ≥ 7 weeks; in contrast, the present study employed C57BL/6J mice aged 6weeks and aggressors aged 10–12 weeks. Furthermore, corticosterone was measured on SDS Day 10 in the former, while here it was assessed on Day 11. Importantly, avoidance behavior identified during SIT in the previous study was not evident in our results. These variations may stem from differences in the age and strain of subject mice at the time of SDS exposure, the relative age of aggressors, and the timing of hormonal measurement. Younger animals, characterized by heightened neuroplasticity and ongoing development of social cognition, may display distinct behavioral and endocrine responses to SDS. Moreover, as corticosterone levels are known to rise acutely and then decline over time, differences in sampling time could critically impact measured concentrations. Our findings underscore the importance of controlling for age and timing variables in SDS models. Future research should systematically investigate how these factors shape behavioral and endocrine outcomes, as well as assess reproducibility and strain‐specific sensitivity to social stress.

This study had several limitations. First, the effects of SRBT or Flu on other types of stress were not examined, thus limiting the generalizability of the findings to other stress conditions. In addition, C57BL/6J mice were used, but different results could potentially be obtained in other species or strains. Thus, further research is required before these findings can be applied to humans. Furthermore, the sample size was small, which might have affected the statistical robustness of the results. The dosage and duration of SRBT administration were also limited, leaving the effects of higher doses or long‐term administration unexplored. Although a comparison with Flu was conducted, comparing SRBT with other anxiolytics or antidepressants could have provided a more detailed evaluation of its relative effects. Finally, individual differences among mice and variability attributable to environmental factors might not have been sufficiently considered.

## CONCLUSION

This study suggested that SRBT has the potential to mitigate anxiety caused by social stress. However, further ongoing evaluation and investigation are required to assess the effectiveness of SRBT.

## AUTHOR CONTRIBUTIONS

**Yoshikazu Kitai**: Writing—original draft; methodology; investigation; formal analysis; data curation; visualization. **Leo Gotoh**: Conceptualization; methodology; investigation; formal analysis; data curation; writing—review and editing. **Hikaru Hori**: Supervision; project administration; methodology; conceptualization; writing—review and editing.

## CONFLICT OF INTEREST STATEMENT

The authors declare no conflicts of interest.

## ETHICS APPROVAL STATEMENT

All procedures were conducted in accordance with the Fukuoka University Ethical Review Board on Animal Testing (approval number: 2409042).

## PATIENT CONSENT STATEMENT

This is not applicable for that specific section.

## CLINICAL TRIAL REGISTRATION

This is not applicable for that specific section.

## Data Availability

The data that support the findings of this study are available from the corresponding author upon reasonable request.
